# Anthropogenic Halo Disturbances Alter Landscape and Plant Richness: A Ripple Effect

**DOI:** 10.1371/journal.pone.0056109

**Published:** 2013-02-12

**Authors:** Bingliang Liu, Jinbao Su, Jianwei Chen, Guofa Cui, Jianzhang Ma

**Affiliations:** 1 College of Nature Reserve, Beijing Forestry University, Beijing, China; 2 College of Economics and Management, Northeast Forestry University, Harbin, China; 3 Wildlife and Nature Reserve Management Division of State Forestry Administration, Beijing, China; 4 College of Wildlife Resource, Northeast Forestry University, Harbin, China; Monash University, Australia

## Abstract

Although anthropogenic landscape fragmentation is often considered as the primary threat to biodiversity, other factors such as immediate human disturbances may also simultaneously threaten species persistence in various ways. In this paper, we introduce a conceptual framework applied to recreation landscapes (RLs), with an aim to provide insight into the composite influences of landscape alteration accompanying immediate human disturbances on plant richness dynamics. These impacts largely occur at patch-edges. They can not only alter patch-edge structure and environment, but also permeate into surrounding natural matrices/patches affecting species persistence–here we term these “Halo disturbance effects” (HDEs). We categorized species into groups based on seed or pollen dispersal mode (animal- vs. wind-dispersed) as they can be associated with species richness dynamics. We evaluated the richness of the two groups and total species in our experimental landscapes by considering the distance from patch-edge, the size of RLs and the intensity of human use over a six-year period. Our results show that animal-dispersed species decreased considerably, whereas wind-dispersed species increased while their richness presented diverse dynamics at different distances from patch-edges. Our findings clearly demonstrate that anthropogenic HDEs produce ripple effects on plant, providing an experimental interpretation for the diverse responses of species to anthropogenic disturbances. This study highlights the importance of incorporating these composite threats into conservation and management strategies.

## Introduction

Anthropogenic landscape alteration and habitat fragmentation are considered major threats to global biodiversity [1], [2], and they have become critical research fields in conservation [3], [4]. Thus, it is essential to understand the interactions between humans and nature in the process of the evolution of landscapes [5], [6] and how anthropogenic landscape alterations affect species persistence [7].

Seed dispersal largely determines the ability of plants to persist, expand, and colonize habitat [8]; at a local scale, the persistence times of plants are largely dominated by ecological processes operating at short time scales (e.g., dispersal, immigration, population dynamics, and contraction or expansion of species geographic ranges) as local extinctions are dynamically balanced by colonization [9]. Although life-history theory [10] may provide a framework into trade-offs that constrain the movement abilities of species, anthropogenic habitat fragmentation, changes in land-use patterns and climate, and the introduction of exotic species further link organismal movement with environmental variables [11]. External environmental variables such as connectivity, the abundance and behaviour of dispersal vectors, and the quality and quantity of habitat may also strongly interact with life-history traits of plant individuals (such as motion capacity and internal states of pollen, seeds or vegetative tissues), to influence seed movement, plant recruitment, and community composition [12].

Among external environmental variables, connectivity critically determines population survival [13] as it can influence the migration of species [14]. Yet in discussing connectivity, it is important to consider that different organisms may experience the same geographical area in different ways, because of variation in the ways in which life histories interact with the physical organization and properties of habitats in landscapes. Accordingly, connectivity has been distinguished between structural and functional [15]. Structural connectivity refers to landscape patterns, such as the density of corridors [16], distance between patches, and matrix percolation ability [17], [18]. Functional connectivity is defined by the extent to which an individual species of interest can move through a landscape [14]. Functional connectivity is more complex. It depends not only on landscape patterns, but also on the interactions between the patterns and the biological characteristics of a given species [19]. In fact, structurally connected patches still may not be functionally connected and even non-contiguous patches may be functionally connected, depending on the species [20]. Some studies also view connectivity from three aspects–landscape connectivity, habitat connectivity and ecological connectivity [21]. Landscape connectivity may translate into habitat connectivity benefitting some given species but not all species [16]. Ecological connectivity tends to a positive correlation with landscape connectivity [14]. However, enhancing landscape connectivity may not mean increasing all aspects of ecological connectivity [22]. Therefore, the effects of different connectivity on species must be differentiated.

Nathan et al. [11] have proposed a movement ecology framework that connects the basic aspects of life history and the behaviour (internal state, motion ability, navigation capacity) of individuals’ movement with environmental variables such as connectivity, and represents all aspects of the abiotic and biotic environment influencing species movement. Previous literature has brought to light valuable insight for research on the effects of fragmentation on seed dispersal or mutualisms between given species [8], [23]–[26]. Yet, human footprints now permeate most landscapes worldwide. In addition to the chronic influences of fragmentation, other anthropogenic disturbance may simultaneously affect species persistence in various ways. For example, noise pollution alters ecological services such as seed dispersal by affecting ecological mutualisms [27], [28]. These composite threats should be considered jointly.

Recreation landscape (RL), may supply a suitable case for studying on the composite effects on species persistence as it involves both the chronic influences arising from landscape modification and immediate human disturbances. This class of landscape consists of recreation patches, recreation corridors (traffic, roads etc.) and natural matrices/patches [29]. Recreation patches and travel corridors are commonly partially deforested of native vegetation for the building of recreational facilities and grounds; the formerly continuous natural landscapes are likely to be divided into clusters, leading to the alteration of the landscape in structure and pattern. This often reduces habitat connectivity while conversely increasing landscape connectivity. More importantly, this type of landscape, with intricate patterns and ecological processes [30], is frequently permeated with anthropogenic high-frequency disturbances (activity disturbances, noise disturbances, etc.).

**Figure 1:**
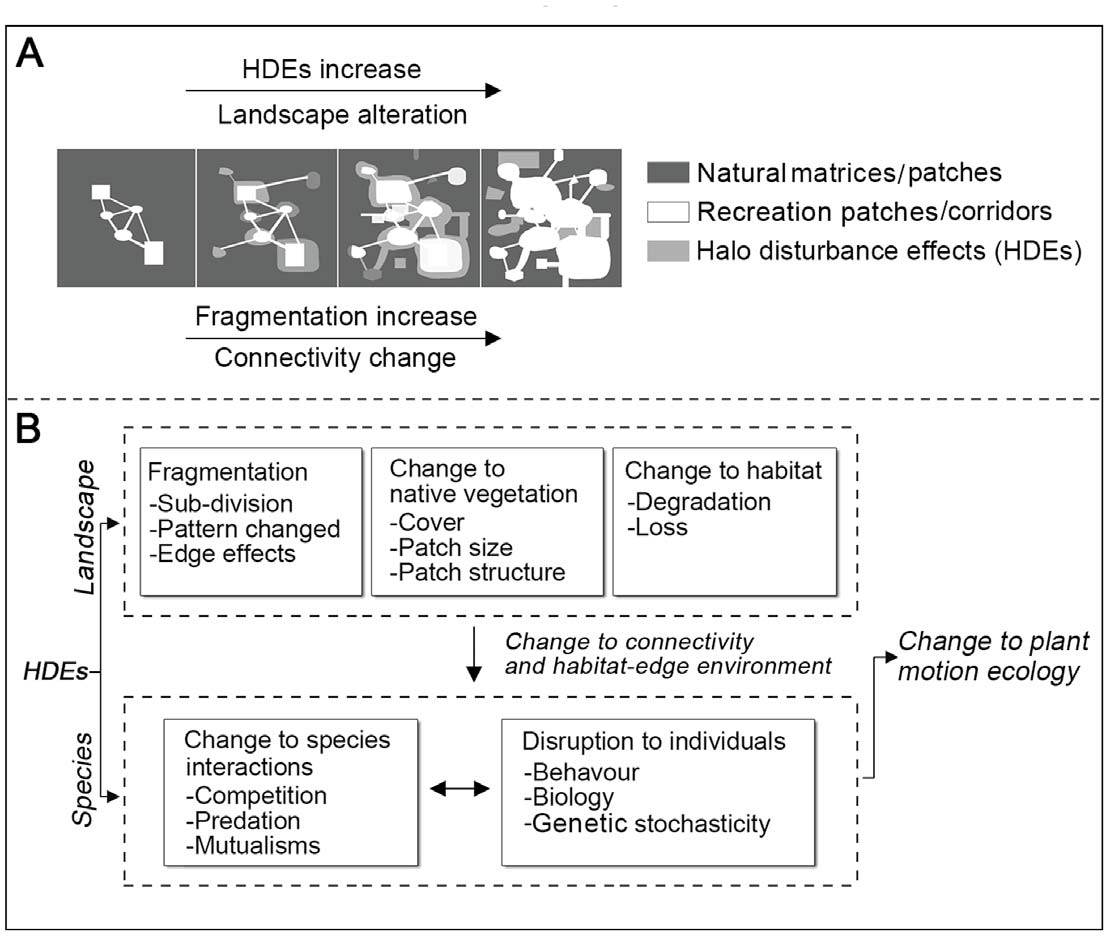
Conceptual framework of “Halo disturbance effects (HDEs)”. *A*, prediction model of recreation landscape (RL) fragmentation (modified from [35]). *B*, HDEs can not only modify the structure and pattern of landscapes leading to the reduction of habitat area and the alteration of connectivity [36], but also produce ripple effects on species by disrupting mutualisms between species (modified from [37]).

Generally, effects on landscapes and species principally result from the alteration of circumstances of fragmented boundaries in both physical and biological ways as edge-effects can considerably affect species richness, composition, community dynamics, and ecosystem functionality [31]–[34]. In RLs, while anthropogenic disturbances similarly frequently occur at patch-edges, they can not only alter patch-edge structure and environment, but also permeate into surrounding natural matrices/patches affecting species persistence–here we term these “Halo disturbance effects” (HDEs) – they increase the area exposed to edge effects and as a consequence, an intact landscape is divided into clusters leading to its fragmentation (Figure 1). Fragmentation may decrease the functional connectivity of the landscape, threatening the viability of sensitive species [38]. In this case, HDEs can be understood on two levels: large-scale chronic disturbances (landscape modification) and small-scale immediate disturbances (e.g., threats from human behaviour and noise pollution). The conceptual framework may provide a critical insight into the understanding of the interactions between human activities and the evolution of landscapes and how human activities affect plant persistence.

**Figure 2:**
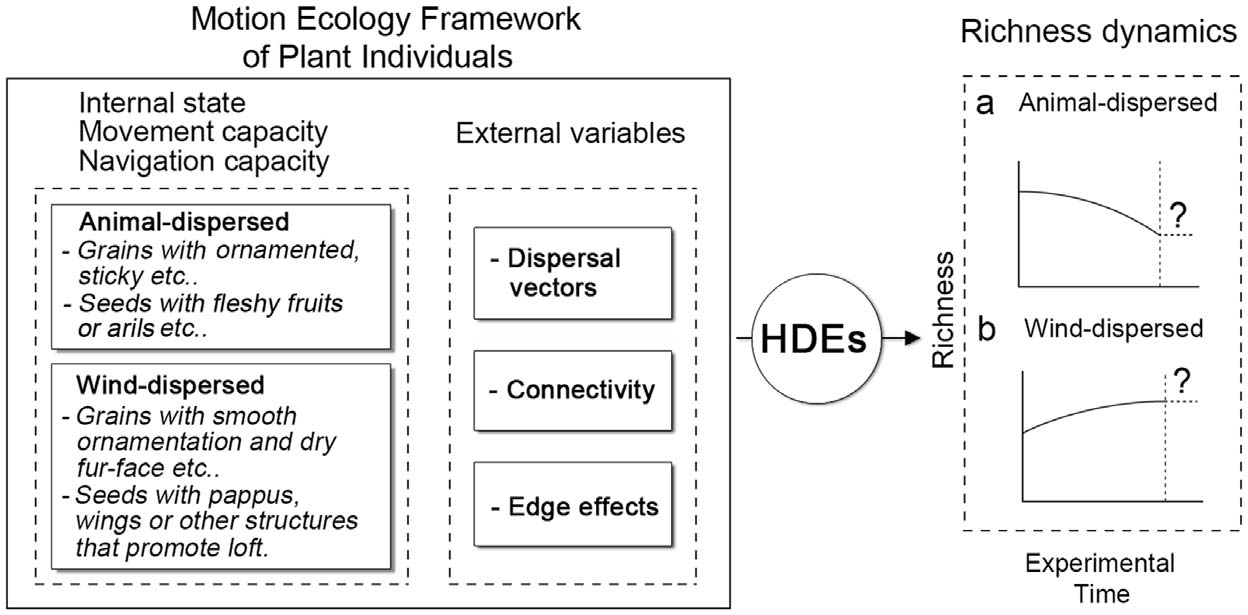
Predictions of animal- and wind-dispersed species in richness dynamic for our experimental landscapes (modified from [**11**], [**12**]). The dashed lines exceed our experimental time and relax to an unknown trend. We cannot make predictions about them.

Based upon the life-history traits of plants [10], the movement ecology framework [11], [12] and connectivity theories [13]–[22], we scale up from plant individuals to total communities by using seed dispersal modes (animal- and wind-dispersed) as a proxy for movement capacity to test anthropogenic HDEs based on the following reasons: (1) the two groups largely contribute to plant community dynamics; (2) dispersal vectors (e.g., animals, wind) are related to the movement of plants as they can assist plant spread in different ways [11]; and (3) HDEs may interact with internal state of plants such as seed size by altering external factors of the movement ecology framework such as connectivity, edge-effects, dispersal vector’ activities etc. to influence plant persistence. We predict that animal- and wind-dispersed species may have different responses to HDEs in our experimental landscapes disregarding other variables (weather events, natural catastrophes and stochastic factors from demography and genetics etc.): animal-dispersed species will be negatively affected by HDEs through ecological effects on animal communities and disruption to the mutualisms of seed dispersal (e.g., refs. [8], [21]), while wind-dispersed species may respond positively as they can benefit from wind dynamics in open landscapes [12] and the alteration of edge-habitat in environments [39] (Figure 2). We also predict that landscapes with different intensity of human use will have different responses in richness.

To test these predictions, we need to answer 3 questions. First, how do HDEs occur? Second, how do they affect landscapes and species richness? And third, how can we reduce this effect for biodiversity conservation and management? To achieve this, we first offered a preliminary test for the richness of each group (animal-dispersed species, wind-dispersed species and total species) between the years assessed. We then assessed the difference in intensity of human use in our experimental RLs. We finally discussed the mechanisms of HDEs on species persistence and how to reduce these effects for biodiversity conservation and management.

## Materials and Methods

### Ethics Statement

This study obtained relevant permissions from reserve administrations and was conducted under the Wildlife Protection Law of the People’s Republic of China.

**Figure 3:**
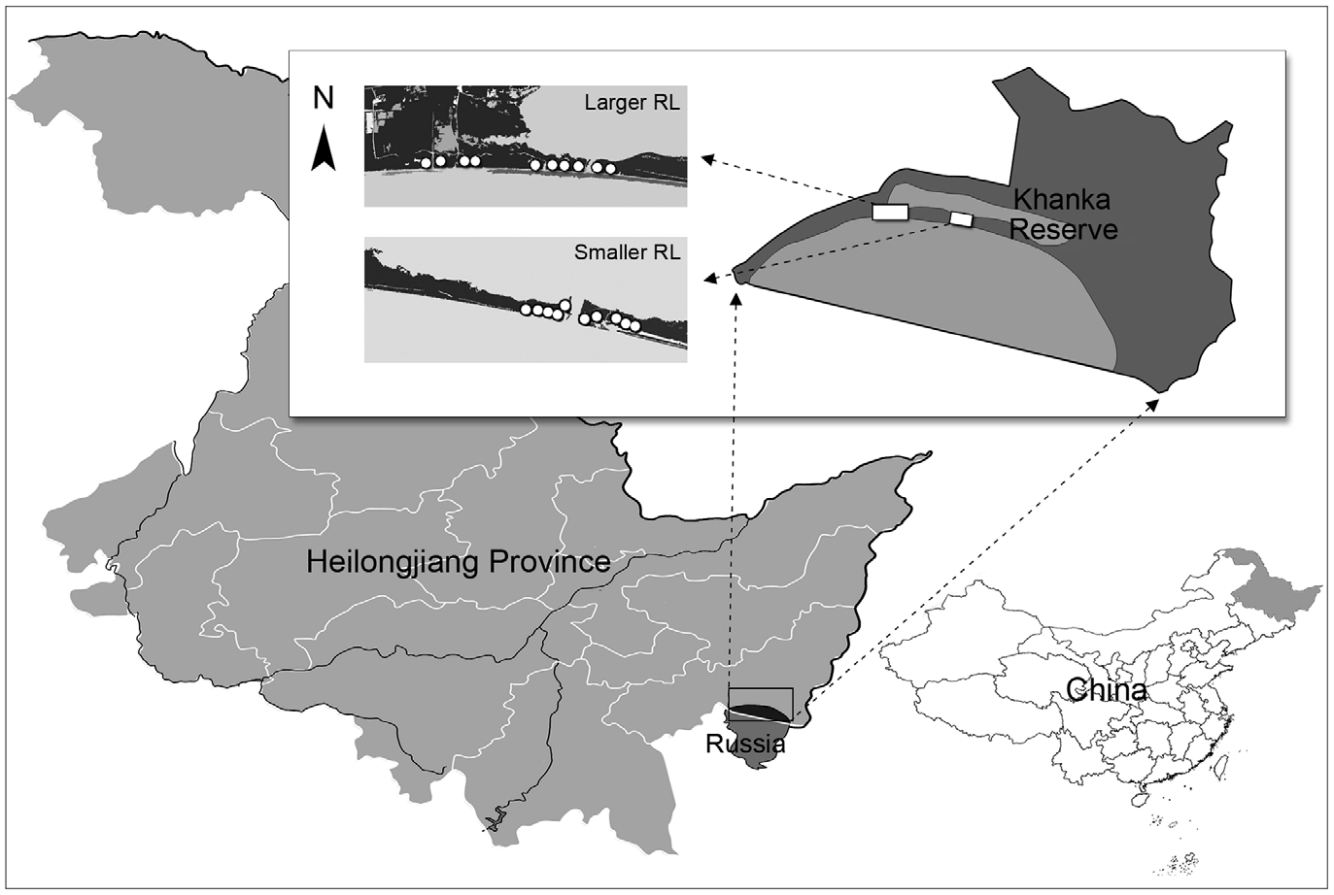
Study locations within the Khanka Reserve. This Reserve lies at the boundary of Heilongjiang Province, China. Each point represents one study transection.

The study was conducted in Khanka Nature Reserve (45°01′ to 45°34′N, 131°58′ to 133°07′E). This reserve lies at the boundary of Heilongjiang Province, China (Figure 3), and the Chinese-Russian border runs through it. The portion of China with most of bottomlands and denes has an area of 222,488 ha. It is a typical temperate monsoon climate and has an average annual precipitation of 654 mm, wind speed of 4.0 m/s (the max. 17.5 m/s) and temperature of 3°C. This reserve is a pivotal area for millions of migratory birds. Some of the nationally endangered plant species include *Pinus takahasii Nakai*, *Juglans mandshurica, Fraxinus mandschurica, Tilia amurensis, Glycine soja, Nelumbo nucifera* etc. Of them, *Pinus Takahasii Nakai* is the endemic endangered species in the reserve.

The large scale tourism development in the reserve was initiated from 2005. In order to test the responses of species to human disturbance, we took advantage of that to design a before-and-after monitoring from June 2006 to September 2011. We selected two RLs with different development scales within a lakefront forest to conduct our study. The larger one (approx. 20 ha) has a higher intensity of human use than the smaller one (approx. 10 ha). They are homogeneous in geology, geomorphology, climate and environment. Within each landscape, we established 10 permanent transects that were perpendicular to the edges of recreation patches and corridors. Each of them was subdivided into 5 contiguous 10×10 m plots [23]. We conducted an annual census of all vascular plant species occurring in each plot across our experimental period. Species were identified from samples and photographs taken in the field, compared with Flora of China, and verified by regional botanical experts. We divided them into two groups (animal-dispersed and wind-dispersed) based on previously known information or using seed morphological characteristics for unknown species. For those external (outside experimental plots) species with gravity- or water-dispersed mode that could not contribute to the rapid change of the local community within the time scale of our study [12], we treated them as a null model. For native species (within experimental plots) with ballistic, gravity or water dispersal modes might also be affected by HDEs through the disruption of pollination or the prevention of seedling-emergence, we categorized them into an animal- or wind-dispersed group depending on their pollination mode (Table S1).

All subsequent analyses were based on plot-level data. To accomplish these goals, we selected the *Gleason-richness* index to test the mechanism of HDEs on plant richness. To support our conclusions, we created 3 fitting models with Origin-pro software (Version 8.0; OriginLab Development) respectly for each group in the two RLs to interpret their richness dynamics across distances from patch-edge. We used multiple comparisons (ANOVA) (with significance at *P*<0.05) to test for the significance of the spatial difference in each group respectively in 2006 and 2011. By using paired-sample *t*-test (with significance at *P*<0.05), we tested for the level of richness change over years. We also compared the average change rates of each group’ richness in our experimental landscapes. We utilized intensity of human use (e.g., numbers/100 m^2^) as a proxy for the estimation of HDEs. These tests were suitable for the assessment of species in responses to HDEs in different spatial scales between the years assessed.

## Results

Throughout the study period, a total of 129 plant species were categorized into two groups (67 animal-dispersed species; and 62 wind-dispersed species) according to our study model. Mean values across years were 93.1 species (53.3 animal-dispersed species; 39.8 wind-dispersed species) for the larger RL and 87.4 (52 animal-dispersed species; 35.4 wind-dispersed species) for the smaller RL.

**Figure 4:**
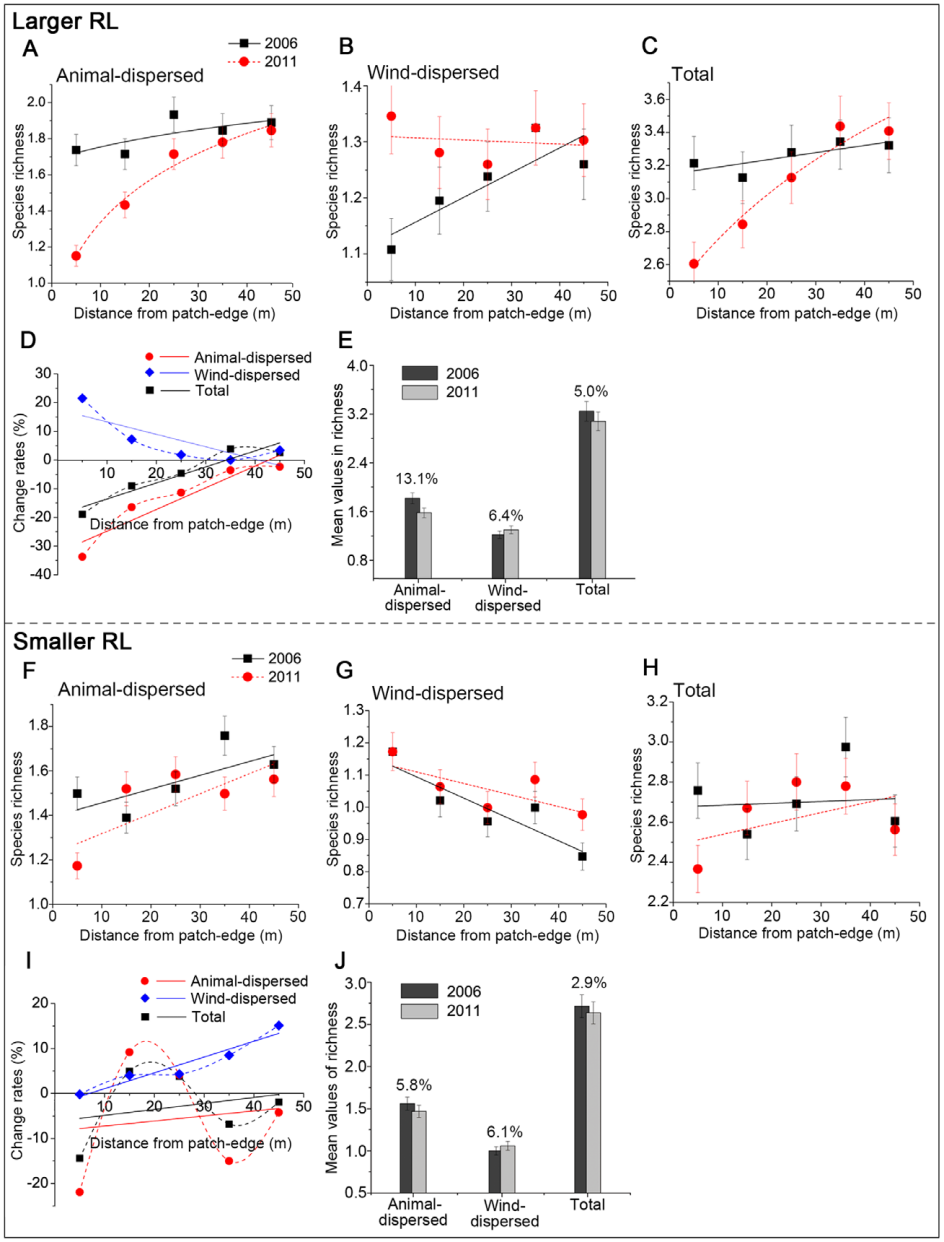
Species richness of animal- and wind-dispersed groups, and total communities. *A–C* and *F–H* represent average species richness at each distance in the two landscapes; *D* and *I* represent the change rates of each group at each distance; *E* and *J* represent overall change of each group in richness across distances from patch-edge.

In 2006, across distances from patch-edge, each group in both larger RL (animal-dispersed: F = 0.868, *P*>0.4; wind-dispersed: F = 1.035, *P*>0.3; total: F = 0.405, *P*>0.8) and smaller RL (animal-dispersed: F = 1.458, *P*>0.2; wind-dispersed: F = 2.45, *P*>0.05; total: F = 1.271, *P*>0.2) did not reveal spatial significant differences in richness. Consistent with our predictions, over the 6 years, both animal- and wind-dispersed species had responses to HDEs and that led to changes in total richness in the two RLs (Figure 4 and Table S2).

In the larger RL, HDEs produced profound negative effects on animal-dispersed species at all distances over years (Figure 4A), and richness decreased by an average of 13.1% (t = −2.441, df = 4, *P*>0.07; Figure 4E). The most significant rate of decay occurred at a distance of 0 to 10 m, reaching 33.7% (t = −7.356, df = 9, *P*<0.001). Yet at distances further within the patch such as 30 to 40 m (t = −0.428, df = 9, *P*>0.6) and 40 to 50 m (t = −0.391, df = 9, *P*>0.7), richness did not notably decay (Figure 4 A and D; Table S2). Over years, HDEs led to a significant gradient effect across distances from patch-edge (F = 10.3, *P*<0.001; Figure 4A) in animal-dispersed species. In contrast, HDEs positively affected wind-dispersed species, and richness had an average elevation of 6.4% (t = 1.831, df = 4, *P*>0.1; Figure 4 B and E) over years. This group increased significantly near patch-edge (0 to 10 m: t = 4.714, df = 9, *P*<0.005), but we found no evidence for a change at a distance of 30 to 40 m (t = 0.000, df = 9, *P* = 1.000). Despite the fact that in 2011 this group did not reveal spatial significant differences (F = 0.23, *P*>0.9) in richness from 0 to 50 m, its rates of change generally exhibit a negative correlation with distance from the patch-edge (Figure 4D).

In this larger RL, although wind-dispersed species were augmented over years, total richness had a decay of 5.0% (t = −1.229, df = 4, *P*>0.2; Figure 4 C and E). In 2011, total species richness revealed a significant spatial gradient across distances from patch-edge (F = 9.475, *p*<0.001; Figure 4C), which was driven by the dynamic of the animal-dispersed group (correlations: r = 0.961, *P<*0.01).

Mirroring the patterns of the larger RL, the three groups in the smaller RL were similarly influenced by HDEs over years (Figure 4 F–J). Although animal-dispersed species similarly presented negative responses to HDEs as a whole, relative to the larger RL, they had a decay of only 5.8% across distances (t = −1.03, df = 4, *P*>0.3). This is because the richness of this group had somewhat increased from 10 to 30 m (10 to 20 m: t = 1.203, df = 9, *P*>0.2; 20 to 30 m: t = 0.486, df = 9, *P*>0.6; Figure 4F), leading to a fluctuatuation at different directions across distances from patch-edge (Figure 4I). After 6 years, HDEs enhanced the spatial difference of this group in richness from 0 to 50 m (F = 3.822, *P*<0.05). For wind-dispersed species, HDEs similarly elevated their richness over time (t = 2.737, df = 4, *P*>0.05). The richness of this entire group was up by 6.1% (Figure 4J). Yet relative to the larger RL, the group in the smaller one has a contrary trend in rates of change across distances from patch-edge (Figure 4I): richness increased considerably (t = 1.615, df = 9, *P*>0.1) in the patch-inner (40 to 50 m), while not changing at patch-edge (0 to 10 m).

In the smaller RL, the fluctuation of the animal-dispersed group at different directions contributed to an average decay of only 2.9% (t = −0.798, df = 4, *P*>0.4; Figure 4 H and J) in total richness over years, and thus relative to the larger RL, the smaller one did not reveal a significant spatial gradient (F = 1.857, *P*>0.1) across distances from patch-edge in 2011.

## Discussion

Our findings clearly reveal that species with different dispersal-modes had different responses to HDEs: animal-dispersed species considerably decreased, whereas wind-dispersed species increased. This response was more evident in the larger RL than in the smaller one. These results generally conform to our predictions, indicating that HDE can be interpreted by considering the plant dispersal mode regardless of other factors.

For animal-dispersed species, unsuitable habitats were a primary contributor to species decay, particularly at the distance of 0 to 10 m (Figure 4). Anthropogenic HDEs have destroyed considerable amounts of edge-habitats, leading to artificial edge-effects (e.g., soil drought and desertification, degradation and loss of habitats) and the reduction of habitat connectivity. Some species could be limited by unsuitable micro-sites or seed mortality [40]. However, the loss of species in undegraded habitats may be because HDEs led to ripple effects by disrupting key animal-plant mutualisms. That is, HDEs might have triggered the loss of one or more species which in turn triggered the elimination of other species [41]. Even substantial populations are also likely to become extinct owing to loss of key mutualisms [42]. In our experimental landscapes, too many man-made recreation facilities (more than 100), travel corridors (a total length of more than 60 km) and forest-breaches have resulted in serious landscape fragmentation. This might reduce functional connectivity [38] affecting ecological processes [43] because fragmentation can not only decrease pollinator-visitation leading to pre-dispersal failure [44], but also shorten the distances of seed dispersal by restricting ranges of animal motion [45]. Compared with wind-dispersed species, animal-dispersed species have a high risk of failure in pollination, either through pollinator (e.g., insects, wasps) loss or reduction in abundance [44]. Specialized pollinators may be particularly limited in small fragments [46]. In addition to landscape modification, immediate HDEs could have disrupted animals interacting with plants, reducing the probability of their assistance in seed dispersal [36]. For example, noise may threaten some fruit-eating animals [25] especially birds [28]. Because some birds with specialized vocal-frequencies fail to communicate with each other in the presence of the similarly-frequency noise, they may have flown from noisier areas to quieter ones [47].

Rarity (low abundance within a habitat) may be a particular concern for susceptibility to extinction [48]. In our experimental RLs, small or sparse populations of most animal-dispersed species likely led to a low probability of pollination success due to the lack of potential mates and thus resulted in a failure to replace themselves [49]. Small or sparse populations can also decrease the threshold distance for pollination success of native animal-pollinated species [50].

For wind-dispersed species, they increased overall in both RLs (Figure 4 E and J). This conforms to our prediction, supporting hypothesis that changes of landscape in structure and pattern both increase ecological connectivity facilitating seed dispersal by wind (e.g., refs. [12], [40], [51]) and supply space for the introduction of species [40]. The intrusion of large numbers of visitors and vehicles might also be a contributor to the introduction of some exotic species. Our records based on plots show that more than 80% of the exotic species were weed species with abundant tiny seeds, which conform to our rudimentary understanding about the relationship between plant internal state such as seed size and morphologies, and external variables such as wind dynamics and landscape connectivity (Figure 2). The establishment of these might have competitively reduced germination and establishment of some low-abundance, animal-dispersed species.

The change of each group in the larger RL is more remarkable than that in the smaller one (Figure 4 E and J). It is not surprising and conforms with our predictions as the larger RL was exposed to a higher intensity of human use than the smaller one (Figure S1), and thus could lead to stronger HDEs. For animal-dispersed species, larger HDEs and artificial patches might jointly lead to a larger isolation distance and they failed to disperse between natural patches separated by very large distances [12]. For wind-dispersed species, the larger RL could supply more adequate space for the introduction of seeds assisted by wind.

Another striking result is that, animal- and wind-dispersed species presented diverse dynamics at different distances from patch-edge in the two landscapes respectively (Figure 4 D and I). In the larger RL, the rates of change in animal-dispersed species declined with distance from patch edge and led to a sharp gradient effect in 2011, indicating that human activities have produced HDEs on species and they are stronger at patch-edges. In our experimental landscapes, decays of edge-habitats in quality and connectivity, the immediate noise disturbance, etc. might have jointly affected the establishment and recruitment of animal-dispersed species. However, in the smaller RL, animal-dispersed species had increased somewhat at distances of 10 to 30 m (Figure 4F). It might attribute to two contrary mechanisms: relatively low HDEs led to a small threshold distance; landscape connectivity in structure facilitated the spillover of some species from inner-habitats towards edge-habitats [23]. As for wind-dispersed species, they show contrary trends in the rate of change across distances from patch-edge (Figure 4 D and I). Species in the larger RL exhibited stronger edge effects, whereas those in the smaller RL exhibited evident connectivity effects. These diverse dynamics are potentially because the changes of the landscapes in structure and pattern altered wind circulation patterns [52], [53], leading to highly variable wind directions, changing dispersal directions for the wind-dispersed seeds [54], as wind dynamics are related to patch geometry [12].

Our findings differ from those landscapes without human disturbances, where landscape connectivity in structure may augment the richness of both animal- and wind-dispersed species in edge-habitats [23]. Yet our results reveal that, in landscapes with high-intensity anthropogenic disturbances, the richness of animal-dispersed species at patch-edges was depressed by anthropogenic HDEs. These findings clearly demonstrate that human activities are potentially impacting innumerable species interactions both directly and indirectly, indicating the necessity for a more mechanistic understanding of the responses of species to human disturbance.

Although we were unable to track the fate of seeds due to the lack of community-wide data (e.g., seed rain and seed banks) which limits our ability to understand the exact underlying mechanisms driving our observed patterns, our concentration was focused on the interactions between species richness dynamics and external environmental factors. The categorization of plants might provide insight into the dynamics of entire communities [12]. Admittedly, the materials we chose for our experiment may be limited and controversial as other factors such as extreme weather events and stochastic factors could produce similar results over time, but we do not see compelling evidence for this. Further research into community-wide dynamics of seed production, frugivore activity and seed dispersal may provide insight into the mechanisms underlying our observed results.

This conceptual framework provides an experimental interpretation in the responses of species to anthropogenic disturbances in RLs. The responses of species are almost certainly linked to the scale, patterns and structure of landscapes. The relative magnitude of HDEs would likely vary by the ranges and intensities of human use. Our results underscore that this effect is a strong environmental force that may alter key ecological processes and services. Conservation may be more effective if incorporating these threats into conservation and management strategies. This leads to following recommendations: (1) stemming landscape modification for the most animal-dispersed species within a natural landscape is crucial for conservation; (2) fencing recreation patches to prevent HDEs or reducing the scales of RLs to maintain the larger patches of undisturbed habitats; (3) reserving adequate buffer zones for the reduction of HDEs may be an essential aspect in landscape development, and (4) promoting the movement of species at patch-edges or buffer zones of reserves by increasing connectivity between suitable patches in various ways. We hope that this conceptual framework will offer a new insight for landscape development and land-management, and stimulate further research on species persistence and community dynamics in areas with rich biodiversity accompanied by a high intensity or frequency of human disturbance.

## Supporting Information

Figure S1 **Intensity of human use in the two experimental recreation landscapes (RLs).** The data were from historical statistics and our random assessment.
(DOC)

Table S1 **Identification and categorization of species.**
(DOC)

Table S2 **Average richness and change rates of animal- and wind-dispersed groups and total communities.**
(DOC)
