# Supplementary figures and images for: Anthropogenic Halo Disturbances Alter Landscape and Plant Richness: A Ripple Effect
Source: PLoS One. 2013 Feb 12;8(2):e56109. doi: 10.1371/journal.pone.0056109 (PMC3570462; doi:10.1371/journal.pone.0056109)

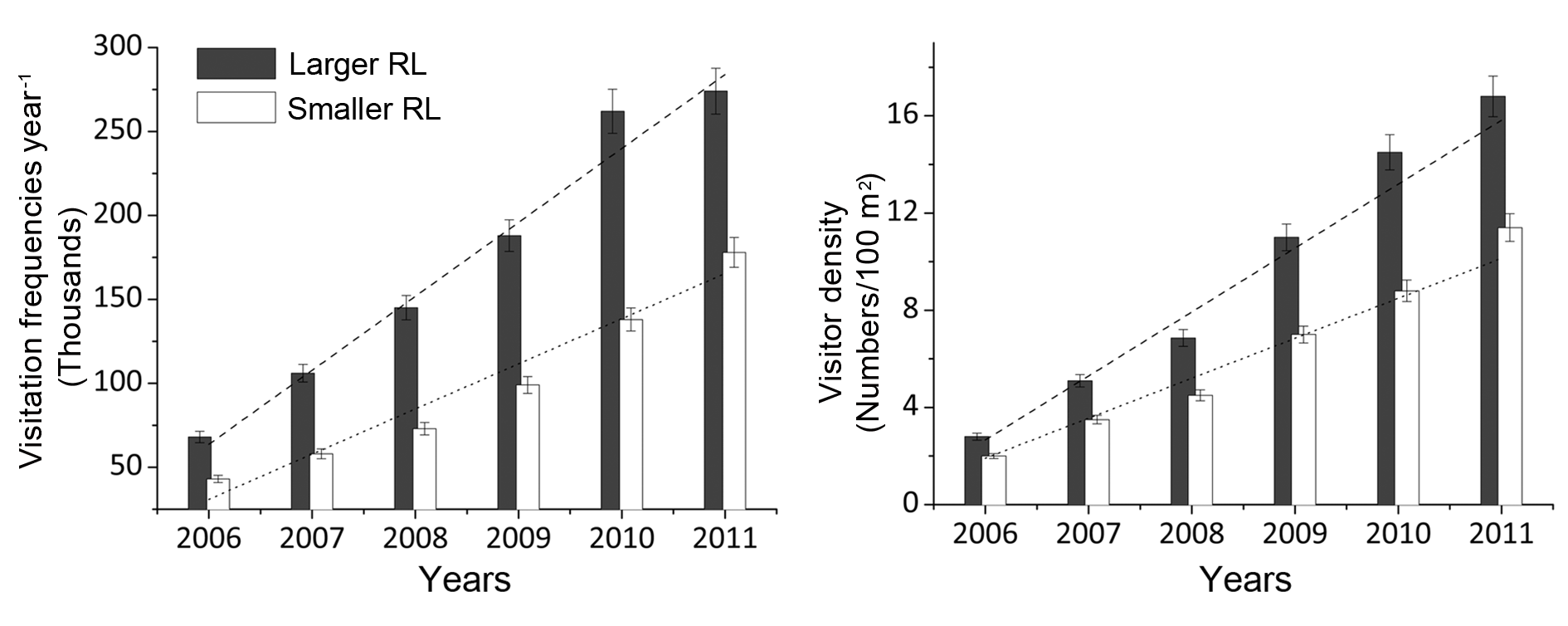
­

Supplement: Figure S1 — Intensity of human use in the two experimental recreation landscapes (RLs). The data were from historical statistics and our random assessment. (DOC) [file pone.0056109.s001.doc]
