# Supplementary material for: Anthropogenic Halo Disturbances Alter Landscape and Plant Richness: A Ripple Effect
Source: PLoS One. 2013 Feb 12;8(2):e56109. doi: 10.1371/journal.pone.0056109 (PMC3570462; doi:10.1371/journal.pone.0056109)
Supplement: Table S1 — Identification and categorization of species. (DOC) [file pone.0056109.s002.doc]

**Table S2 Identifying and categorizing of species.**

|  |  |  |
| --- | --- | --- |
| **Dispersal mode** | **Family** | **Species** |
| **Animal-dispersed** | Araliaceae | *Eleutherococcus senticosus* |
|  |  | *Eleutherococcus sessiliflorus* |
|  | Berberidaceae | [*Berberis ferdinandi-coburgii*](http://db.kib.ac.cn/eflora/view/search/Chs_contents.aspx?CPNI=CPNI-142-49295) |
|  |  | *Berberis sibirica* |
|  | Caprifoliaceae | *Lonicera ruprechtiana* |
|  |  | *Lonicera chrysantha* |
|  |  | *Viburnum burejaeticum* |
|  |  | *Sambucus williamsii* |
|  | Celastraceae | *Euonymus alatus* |
|  |  | *Euonymus verrucosus* |
|  |  | *Euonymus echinatus* |
|  | [Compositae](http://www.nature-museum.net/920.spid) | [*Xanthium sibiricum*](http://www.nature-museum.net/43787.spid) |
|  |  | Lactuca sativa |
|  |  | Serratula coronata |
|  |  | [*Parasenecio hastatus*](http://www.nature-museum.net/40655.spid) |
|  |  | Aster tataricus |
|  | [Convolvulaceae](http://www.nature-museum.net/889.sp) | Calystegia hederacea |
|  | Fagaceae | *Quercus mongolica* |
|  | Fabaceae | [*Glycine soja*](http://www.nature-museum.net/23316.sp) |
|  |  | Lespedeza bicolor |
|  |  | [*Vicia amoena*](http://www.nature-museum.net/24386.sp) |
|  |  | [*Vicia cracca*](http://www.nature-museum.net/24373.sp) |
|  |  | [*Vicia pseudorobus*](http://www.nature-museum.net/24383.spid) |
|  |  | [*Vicia amurensis*](http://www.nature-museum.net/24392.sp) |
|  |  | Lathyrus vaniotii |
|  |  | [*Lathyrus humilis*](http://www.nature-museum.net/24436.spid) |
|  |  | Melilotus albus |
|  |  | Melilotus officinalis |
|  | Geraniaceae | [*Geranium wilfordii*](http://www.nature-museum.net/29035.sp) |
|  |  | *Geranium pratense* |
|  |  | [*Geranium krameri*](http://www.nature-museum.net/29075.spid) |
|  |  | Geranium platyanthum |
|  | Juglandaceae | [*Juglans mandshurica*](http://www.plants.csdb.cn/eflora/view/search/Chs_contents.aspx?CPNI=CPNI-254-04027) |
|  | Liliaceae | *Convallaria majalis* |
|  |  | *Asparagus dauricus* |
|  |  | *Polygonatum sibiricum* |
|  |  | *Polygonatum odoratum* |
|  |  | *Polygonatum involucratum* |
|  |  | *Polygonatum humile* |
|  |  | *Paris verticillata* |
|  |  | *Smilacina trifolia* |
|  |  | *Smilacina davurica* |
|  | [Paeoniaceae](http://www.nature-museum.net/969.spid) | [*Paeonia lactiflora*](http://www.nature-museum.net/13397.spid) |
|  | Ranunculaceae | *Anemone rivularis* |
|  | Rhamnaceae | *Rhamnus davurica* |
|  |  | *Rhamnus diamantiaca* |
|  |  | *Rhamnus parvifolia* |
|  |  | *Rhamnus ussuriensis* |
|  | Rosaceae | *Malus baccata*  [*Rosa davurica var. davurica*](http://www.nature-museum.net/721803.sp) |
|  |  | [*Rosa koreana*](http://www.nature-museum.net/21881.spid) |
|  |  | *Rosa acicularis* |
|  |  | *Crataegus pinnatifida* |
|  |  | *Crataegus maximowiczii* |
|  |  | *Geum aleppicum* |
|  |  | *Sorbus pohuashanensis* |
|  | Rubiaceae | [*Rubia cordifolia*](http://www.nature-museum.net/38408.sp) |
|  |  | [*Rubia chinensis*](http://www.nature-museum.net/38390.sp) |
|  | Rutaceae | *Dictamnus dasycarpus* |
|  | Schisandraceae | *Schisandra chinensis* |
|  | Saxifragaceae | *Ribes maximowiczianum* |
|  |  | *Ribes mandshuricum* |
|  |  | *Ribes ussuriense* |
|  | Solanaceae | [*Solanum nigrum*](http://www.nature-museum.net/31818.spid) |
|  | Umbelliferae | [*Torilis scabra*](http://www.nature-museum.net/29713.sp) |
|  | [Iridaceae](http://www.nature-museum.net/668.spid) | [*Iris uniflora*](http://www.nature-museum.net/50686.spid) |
|  | [Vitaceae](http://www.nature-museum.net/811.spid) | *Vitis amurensis* |
| **Wind-dispersed** | Aceraceae | *Acer mono* |
|  | Amaranthaceae | [*Amaranthus retroflexus*](http://www.nature-museum.net/12089.sp) |
|  | Athyriaceae | *Athyrium brevifrons* |
|  | Betulaceae | *Alnus fruticosa var. mandshurica* |
|  | Cyperaceae | *Carex ussuriensis* |
|  |  | *Carex lanceolata* |
|  |  | *Carex pilosa* |
|  |  | [*Carex schmidtii*](http://www.nature-museum.net/46431.spid) |
|  |  | *Eriophorum vaginatum* |
|  |  | *Amaranthus hybridus* |
|  | Compositae | *Artemisia viridissima* |
|  |  | *Artemisia argyi* |
|  |  | *Artemisia carvifolia* |
|  |  | *Artemisia lavandulaefolia* |
|  |  | [*Artemisia integrifolia*](http://www.nature-museum.net/42844.spid) |
|  |  | *Atractylodes japonica* |
|  |  | *Taraxacum ohwianum* |
|  |  | [*Saussurea amara*](http://www.nature-museum.net/39361.spid) |
|  |  | [*Saussurea parviflora*](http://www.nature-museum.net/39572.spid) |
|  |  | *Erigeron acer* |
|  | Crassulaceae | *Orostachys minutus* |
|  | [Cruciferae](http://www.nature-museum.net/1322827.sp) | *Cardamine parviflora* |
|  |  | *Dontostemon micranthus* |
|  |  | *Draba nemorosa* |
|  |  | *Lepidium ruderale* |
|  | [Caryophyllaceae](http://www.nature-museum.net/730.sp) | *Stellaria media* |
|  | Chenopodiaceae | *Atriplex patens* |
|  | [Euphorbiaceae](http://www.nature-museum.net/789.spid) | *Euphorbia lucorum* |
|  | [Equisetaceae](http://www.nature-museum.net/519.sp) | *Equisetum pratense* |
|  |  | *Eguisetum sylvalicum* |
|  | Gramineae | *Echinochloa crusgalli* |
|  |  | *Deyeuxia purpurea* |
|  |  | *Deyeuxia langsdorffii* |
|  |  | *Arundinella anomala* |
|  |  | *Roegneria ciliaris* |
|  |  | *Calamagrostis turczaninowii* |
|  |  | *Setaria viridis* |
|  | Gentianaceae | *Gentiana cruciata* |
|  | Liliaceae | *Veratrum nigrum* |
|  | [Labiatae](http://www.nature-museum.net/1322848.sp) | Prunella vulgaris  [*Phryma leptostachya subsp. asiatica*](http://www.nature-museum.net/34425.spid) |
|  | Lycopodiaceae | *Lycopodium clavatum* |
|  | Oleaceae | *Syringa reticulata var. mandshurica* |
|  | Plantaginaceae | *Plantago asiatica* |
|  | Portulacaceae | *Portulaca oleracea* |
|  | Polygonaceae | *Rumex acetosa* |
|  |  | [*Polygonum aviculare*](http://www.nature-museum.net/13023.sp) |
|  | Pteridiaceae | *Pteridium aquilinum var.latiusculum* |
|  | [Pinaceae](http://www.nature-museum.net/599.sp) | *Pinus takahasii Nakai* |
|  |  | *Pinus sylvestris var. mongolica* |
|  | [Ranunculaceae](http://www.nature-museum.net/737.sp) | [*Cimicifuga dahurica*](http://www.nature-museum.net/7052.sp) |
|  |  | *Clematis mandshurica* |
|  | [Scrophulariaceae](http://www.nature-museum.net/896.sp) | *Pedicularis verticillata* |
|  | Salicaceae | *Salix linearistipularis* |
|  | [Urticaceae](http://www.nature-museum.net/703.sp) | [*Urtica angustifolia*](http://www.nature-museum.net/10081.sp) |
|  | Umbelliferae | *Saposhnikovia divaricata* |
|  |  | Heracleum hemsleyanum |
|  |  | *Bupleurum chinense* |
|  |  | [*Cnidium monnieri*](http://www.nature-museum.net/30160.spid) |
|  | [Valerianaceae](http://www.nature-museum.net/912.sp) | [*Patrinia rupestris*](http://www.nature-museum.net/38783.spid) |
|  |  | [*Valeriana officinalis*](http://www.nature-museum.net/38800.sp) |
|  | [Violaceae](http://www.nature-museum.net/831.spid) | *Viola philippica* |

**Notes:**

We identified all plant species for dispersal mode by previous preferences or searching regional flora and guides (http://[www.cvh.org.cn](../../../../2-17修改稿/6-6修改稿/Supporting/www.cvh.org.cn)), and consulted Plant Databases [Scientific Database of China Plant Species (DCP) ([http://www.plants.csdb.cn](http://www.plants.csdb.cn/)); Institute of botany, Chinese academy of sciences (http://www.ibcas.ac.cn); BLH-China ([http://www.bhl-china](http://www.bhl-china/). org)]. Dispersal modes not identified by these methods were deduced by seed/fruit or pollen morphology or congener comparison. Zoophilous-general flowers have grains that are ornamented, may be sticky, are 10–300μm in size, can be clumped, and are produced at variable numbers [1,2]. Pollen from wind-pollinated species has smooth ornamentation (e.g., scabrate, microreticulate, microfoveolate, striate), a dry-surface, a size range of 25-40μm, with little (two to three grains) or no clumping, and pollen produced in large quantities and moderate size [1,3]. Animal-dispersed species often have fleshy fruits or fruits with arils, spines, bristles or pricks *etc.*. The seeds from wind-dispersed species have wooly pappus, wings or other structures *etc*.. Gravity-dispersed species have no obvious strucures facilitating to disperse [4]. Ballistically dispersed species were categorized into gravity-dispersed group. For those species with multiple dispersal modes, we treat them by considering primary dispersal syndromes. For unidentified species, we treated them as a null model.

**References**

1. Proctor M, Yeo P, Lack A (1996) The Natural History of Pollination. Portland: Timber Press.
2. Cruden RW (2000) in Pollen and Pollination, eds Dafni A, Hesse M, Pacini E. Springer Wien, New York. pp 143–165.
3. Ackerman JD (2000) in Pollen and Pollination, eds Dafni A, Hesse M, Pacini E Springer Wien, New York. pp 167–185.
4. Damschen EI, Brudvig LA, Haddad NM, Levey DJ, Orrock JL, et al. (2008) The movement ecology and dynamics of plant communities in fragmented landscapes. Proc Natl Acad Sci USA 105: 19078–19083.
