# Supplementary material for: Anthropogenic Halo Disturbances Alter Landscape and Plant Richness: A Ripple Effect
Source: PLoS One. 2013 Feb 12;8(2):e56109. doi: 10.1371/journal.pone.0056109 (PMC3570462; doi:10.1371/journal.pone.0056109)
Supplement: Table S2 — Average richness and change rates of animal- and wind-dispersed groups and total communities. (DOC) [file pone.0056109.s003.doc]

| **Distance** | **Animal-dispersed** | | | | | | **Wind-dispersed** | | | | | | **Total** | | | | | |
| --- | --- | --- | --- | --- | --- | --- | --- | --- | --- | --- | --- | --- | --- | --- | --- | --- | --- | --- |
|  | Larger RL | | Change  rates | Smaller RL | | Change rates | Larger RL | | Change rates | Smaller RL | | Change rates | Larger RL | | Change rates | Smaller RL | | Change rates |
|  | 2006 | 2011 |  | 2006 | 2011 |  | 2006 | 2011 |  | 2006 | 2011 |  | 2006 | 2011 |  | 2006 | 2011 |  |
| ***D_0-10m_*** | 1.737 | 1.151 | -33.7% | 1.498 | 1.173 | -21.7% | 1.108 | 1.346 | 21.5% | 1.173 | 1.173 | 0 | 3.214 | 2.606 | -18.9% | 2.758 | 2.367 | -14.2% |
| ***D_10-20m_*** | 1.715 | 1.433 | -16.4% | 1.390 | 1.520 | 9.4% | 1.195 | 1.281 | 7.2% | 1.021 | 1.064 | 4.2% | 3.127 | 2.845 | -9% | 2.541 | 2.671 | 5.1% |
| ***D_20-30m_*** | 1.933 | 1.715 | -11.3% | 1.520 | 1.585 | 4.3% | 1.238 | 1.260 | 1.8% | 0.956 | 0.999 | 4.5% | 3.279 | 3.127 | -4.6% | 2.692 | 2.801 | 4% |
| ***D_30-40m_*** | 1.846 | 1.781 | -3.5% | 1.759 | 1.498 | -14.8% | 1.325 | 1.325 | 0 | 0.999 | 1.086 | 8.7% | 3.344 | 3.474 | 3.9% | 2.975 | 2.78 | -6.6% |
| ***D_40-50m_*** | 1.889 | 1.846 | -2.3% | 1.629 | 1.563 | -4% | 1.260 | 1.303 | 3.4% | 0.847 | 0.977 | 15.3% | 3.322 | 3.409 | 2.6% | 2.606 | 2.563 | -1.7% |
| ***Mean*** | 1.824 | 1.585 | -13.1% | 1.559 | 1.468 | -5.8% | 1.225 | 1.303 | 6.4% | 0.999 | 1.060 | 6.1% | 3.257 | 3.092 | -5.0% | 2.714 | 2.636 | -2.9% |

Notes: We used *Gleason richness* index to test species richness. *R_gi_= S / lnA. R_gi_*, Gleason richness index; *S*, the numbers of species in each plot; *A*, the area (m^2^) of plot.
